# Microtubule-Associated Protein Mdp3 Promotes Breast Cancer Growth and Metastasis: Erratum

**DOI:** 10.7150/thno.45566

**Published:** 2020-04-12

**Authors:** Tala, Songbo Xie, Xiaodong Sun, Xiaoou Sun, Jie Ran, Linlin Zhang, Dengwen Li, Min Liu, Gang Bao, Jun Zhou

**Affiliations:** 1 State Key Laboratory of Medicinal Chemical Biology, College of Life Sciences, Nankai University, Tianjin 300071, China.; 2 Department of Biomedical Engineering, Georgia Institute of Technology and Emory University, Atlanta, GA 30332, USA.

In the initially published version of this article, the image of the siMdp3#2 group (Hs578T cells, 24 hr) in Figure 3B is wrong. The correct Figure 3B is as follows:

The correction made in this erratum does not affect the original conclusions. The authors apologize for any inconvenience or misunderstanding that this error may have caused.

## Figures and Tables

**Figure 3:**
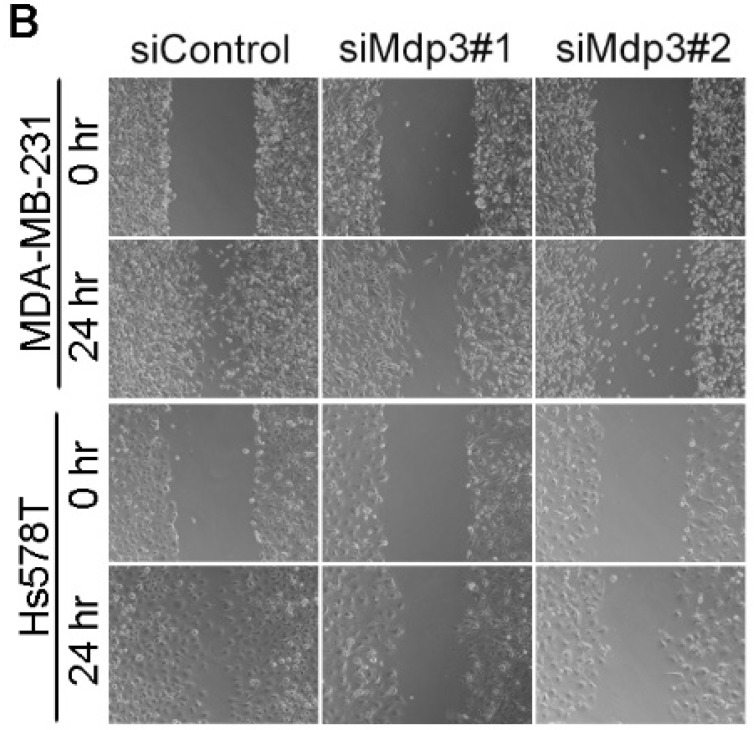
(B) Control or Mdp3 siRNA-transfected cells were scratched, and wound margins were imaged 0 or 24 hours later.
